# PDA (Prolonged Depolarizing Afterpotential) - Defective Mutants: The Story of *nina's* and *ina's—pinta* and *santa maria*, Too

**DOI:** 10.3109/01677063.2011.642430

**Published:** 2012-01-27

**Authors:** William L. Pak, Shikoh Shino, Hung-Tat Leung

**Affiliations:** 1 Department of Biological Sciences, Purdue University, West Lafayette, Indiana, USA; 2 Department of Biological Sciences, Grambling State University, Grambling, Louisiana, USA

**Keywords:** chromophore synthesis, *Drosophila* mutants, forward genetic mutagenesis, PDA-based mutant screen, phototransduction

## Abstract

Our objective is to present a comprehensive view of the PDA (prolonged depolarizing afterpotential)-defective *Drosophila* mutants, *nina*'s and *ina's*, from the discussion of the PDA and the PDA-based mutant screening strategy to summaries of the knowledge gained through the studies of mutants generated using the strategy. The PDA is a component of the light-evoked photoreceptor potential that is generated when a substantial fraction of rhodopsin is photoconverted to its active form, metarhodopsin. The PDA-based mutant screening strategy was adopted to enhance the efficiency and efficacy of ERG (electroretinogram)-based screening for identifying phototransduction-defective mutants. Using this strategy, two classes of PDA-defective mutants were identified and isolated, *nina* and *ina*, each comprising multiple complementation groups. The *nina* mutants are characterized by allele-dependent reduction in the major rhodopsin, Rh1, whereas the *ina* mutants display defects in some aspects of functions related to the transduction channel, TRP (transient receptor potential). The signaling proteins that have been identified and elucidated through the studies of *nina* mutants include the *Drosophila* opsin protein (NINAE), the chaperone protein for nascent opsin (NINAA), and the multifunctional protein, NINAC, required in multiple steps of the *Drosophila* phototransduction cascade. Also identified by the *nina* mutants are some of the key enzymes involved in the biogenesis of the rhodopsin chromophore. As for the *ina* mutants, they led to the discovery of the scaffold protein, INAD, responsible for the nucleation of the supramolecular signaling complex. Also identified by the *ina* mutants is one of the key members of the signaling complex, INAC (ePKC), and two other proteins that are likely to be important, though their roles in the signaling cascade have not yet been fully elucidated. In most of these cases, the protein identified is the first member of its class to be so recognized.

The *nina* and *ina* mutants have played a major role in the elucidation of the *Drosophila* photoreceptor signaling cascade (e.g., reviews: Wang & Montell, 2007; Katz & Minke, 2009; Hardie, 2011). *nina* and *ina* refer to a group of mutants that were identified from alterations in a component of the light-evoked photoreceptor potential known as the “prolonged depolarizing afterpotential” (PDA). For this reason, these have been referred to as the PDA-defective mutants. There are two classes of PDA-defective mutants, *nina* and *ina*, with each class comprising multiple complementation groups. The cellular and molecular functions affected by these mutations are diverse. By and large, the *nina* class of mutants share varying degrees of deficiency in the amount of the major rhodopsin, Rh1. The defects in the *ina* class of mutants are more complex, but their ERG (electroretinogram) phenotypes tend to mimic milder versions of that of the phototransduction channel mutant, *trp* (*transient receptor potential*). Two of the important components of the *Drosophila* phototransduction pathway identified through these mutants include the major rhodopsin, NINAE, the first invertebrate rhodopsin to be molecularly characterized (O'Tousa et al., 1985; Zuker et al., 1985), and INAD, the first scaffold protein identified to orchestrate the formation of a supramolecular signaling complex in a sensory transduction cascade (Huber, 2001; Delmas et al., 2004).

## I. PDA AS A MUTANT SCREENING TOOL

In this review, we will first discuss how we came to use the PDA as a mutant screening strategy and then summarize the information gained from the studies on the *nina/ina* mutants, isolated using this strategy.

### A. The Prolonged Depolarizing Afterpotential (PDA)

**Figure 1:**
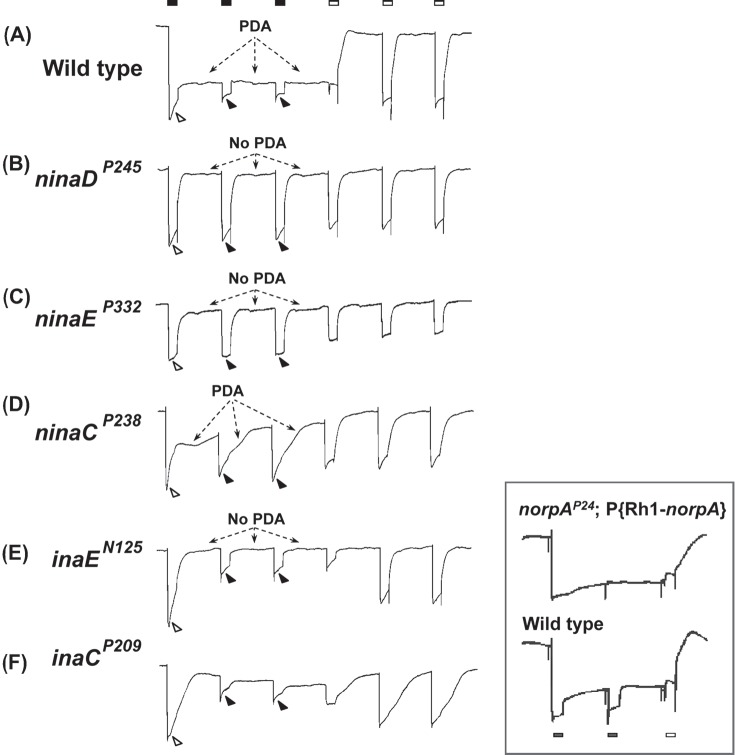
PDA phenotypes revealed in ERG recordings of *nina* and *ina* mutants and wild type. The stimulus protocol is shown at the top: three bright blue stimuli (filled rectangles) each of 4 s duration presented at 20-s intervals followed by three bright orange stimuli (unfilled rectangles) also of 4 s duration presented at 20-s intervals. The first blue stimulus generates a large response that lasts the duration of the stimulus (light-coincident component) in flies of all genotypes (unfilled arrowheads). In wild type, the PDA is generated at the termination of the blue stimulus and maintained throughout the two subsequent blue stimuli (Trace **A**). No PDA is generated in *ninaD^P245^, ninaE^P332^*, or *inaE^N125^* (Traces **B, C,** and **E**), and partial PDAs are generated in *ninaC^P238^* and *inaC^P209^* (Traces **D** and **F**). During the fully developed PDA in wild type, the R1–6 photoreceptors are inactivated, and only small responses originating from R7/8 photoreceptor are elicited by the second and third blue stimuli (Trace **A**, filled arrowheads; also see inset). R1–6 photoreceptors of *inaE^N125^* and *inaC^P209^* are also inactivated by the first blue stimulus and generate only small responses to the second and third blue stimuli (Traces **E** and **F**, filled arrowheads). Thus, in these *ina* mutants, the afterpotential (PDA) is not present, but the R1–6 photoreceptors are inactivated, hence the name *inactivation but no afterpotential*. In strong *nina* mutants, *ninaD^P245^* and *ninaE^P332^*, the PDA is not present and the R1–6 photoreceptors are not inactivated, generating full-amplitude responses to the second and third blue stimuli (Traces **B** and **C**, filled arrowheads). Therefore these mutants were named *neither inactivation nor afterpotential*. The mutant *ninaC^P238^* displays a partial PDA and modest inactivation of R1–6 photoreceptors. The inset illustrates the R7/8 origin of the small responses to the second and third blue stimuli in wild type (Trace **A**, filled arrowheads). It compares the ERG of wild type (bottom) with that of the transgenic fly (top) carrying wild-type *norpA* cDNA driven by Rh1 promotor on a *norpA^P24^* mutant background (*norpA^P24^*; Rh1*-norpA^+^*). The stimulus protocol is shown at the bottom. Since *norpA^P24^* blocks phototransduction and Rh1 drives the expression of wild-type *norpA* cDNA only in R1–6 cells, phototransduction is blocked in R7/8 cells but the block is rescued in R1–6 cells in this transgenic fly. Note that the small response to the second blue stimulus superposed on the PDA is not present in the transgenic fly. This figure was originally published in the *Journal of Biological Chemistry*: Pearn, Randall, Shortridge, Burg, & Pak, 1996, *J Biol Chem, 271*, 4937–4945.

We begin with the discussion of the PDA. In many invertebrate eyes, a colored light stimulus, which favors a substantial photoconversion of rhodopsin to its excited form, metarhodopsin (M^*^), evokes a depolarizing receptor potential that persists in the dark long after the light stimulus is turned off. The PDA refers to this persisting potential in the dark (Figure 1A, arrows). It was first discovered in the lateral ocellus of the barnacle (Hillman et al., 1972) and the median ocellus of *Limulus* (Nolte & Brown, 1972). In rhabdomere-based eyes, the absorption maximum of the visual pigment, rhodopsin (R), is well separated from that of metarhodopsin (M^*^), and, moreover, metarhodopsin is thermally stable at physiological temperatures. In *Drosophila*, the major rhodopsin present in R1–6 photoreceptors, Rh1, and the corresponding metarhodopsin absorb maximally at ∼485 and ∼575 nm, respectively (Ostroy et al., 1974; Pak & Lidington, 1974). Thus it is possible to photoconvert rhodopsin to metarhodopsin and back using blue and orange lights. If a sufficiently bright blue light is used to photoconvert» 20% of rhodopsin to metarhodopsin, the depolarizing potential persists in the dark, i.e., PDA is generated (Figure 1A, arrows); it is then terminated by rephotoconversion of metarhodopsin back to rhodopsin using an orange stimulus (Stephenson et al., 1983) The origin of the PDA is now well established (Dolph et al., 1993). Arrestin 2 (Arr2), one of the two classes of arrestins (Arrestins 1 and 2) present in the *Drosophila* eye, binds to M^*^ to terminate its excitation, but Arr2 occurs at 1/5 the concentration of rhodopsin (LeVine et al., 1990; Matsumoto & Yamada, 1991). If a bright blue stimulus photoconverts Rh1 to M^*^ in molar excess of the available Arr2, M^*^ continues to be active after the cessation of light stimulus and the PDA results. Screening pigments in pigment cells surrounding each ommatidium interfere with the generation of the PDA because they strongly absorb in the blue (Langer, 1967; Strother & Casella, 1972; Stark, 1973). These are genetically removed for ready detection of the PDA.

### B. Adoption of the PDA-Based Mutant Screening Strategy

Keeping in mind that our goal was to isolate a pool of mutants enriched in phototransduction-defective mutants, it is worthwhile reviewing briefly why we decided to use the PDA in our mutant screening protocol in the first place. As we know, the choice of screening strategy is a compromise between two conflicting demands of mutant screening. On the one hand, the strategy must be sufficiently simple to enable a large number of mutagenized flies to be screened rapidly. On the other hand, it must be sufficiently informative to reveal the phenotypes of desired mutants readily.

We started the screen for phototransduction mutants by phototactic behavioral assay mainly because of its simplicity (Pak et al., 1969; review: Pak, 2010). After determining that, with optimization of some of the manipulative steps, ERG recordings could be performed on *Drosophila* relatively simply, we switched to ERG screening to minimize the false positives and negatives inherent in phototactic screening (review: Pak, 2010). Clearly, ERG recordings would more directly test for any defects in photoreceptor response than any behavioral assays, since the main component of the ERG corresponds to the extracellularly recorded, mass response of photoreceptors. However, ERG screening will not select only for phototransduction-defective mutants. The ERG represents the voltage developed across the electrodes due to summed extracellular current flow through the tissues in the head generated by light-evoked responses of all responding cells. Defects in the responses of cells other than photoreceptors could affect the ERG. Moreover, any changes that would alter the passive resistive properties of cells in the current path so as to change either the amount or the paths of extracellular current flow would be reflected in the amplitude and waveform of the ERG, even if they arose from reasons totally unrelated to phototransduction. For example, developmental or degenerative changes in extraretinal neurons or glia in the head could alter the properties of resistive current paths.

In the years following the discovery of the PDA (Hillman et al., 1972; Nolte & Brown, 1972), we became increasingly attracted to it as a potential mutant screening tool that could extend the capabilities of ERG screening. Although the mechanistic understanding of the PDA was rudimentary at the time, it did seem to be closely associated with the photoreceptor potential. If the PDA were an integral component of the receptor potential, any defect in it could be directly attributable to defects in the receptor potential. Moreover, since the PDA is recorded in combination with the “light-coincident” component (Figure 1A, unfilled arrowhead), we thought we could sort out any global effects that affect all components of the ERG, such as those mentioned above, by looking for changes that predominantly or solely affect the PDA with little effect on the light-coincident component.

However, at the time we were considering PDA-based screening, many in the visual physiology community were skeptical whether it represented a physiologically relevant phenomenon at all. If the skeptics were correct, it would be a height of folly to base a major mutagenesis program for the isolation of phototransduction-defective mutants on a phenomenon that has little or no physiological relevance. In 1973, Baruch Minke, who, as a graduate student with Peter Hillman at the Hebrew University, codiscovered the PDA (Hillman et al., 1972), came to us as a postdoctoral associate. Baruch clarified some of the issues we had with the PDA. Although the information was still incomplete, and the mechanistic understanding of the PDA did not come until some two decades later (Dolph et al., 1993), we decided to go with the PDA-based mutant screening strategy anyway. Although we began using the strategy sporadically as early as 1973/74, it was not until the fall of 1975 that we started using it systematically for the isolation of new mutants. In spite of this initial uncertainly, ultimately, the PDA-based screening turned out to be an efficient means of isolating mutants defective in phototransduction and the rhodopsin chromophore synthesis (see Section II).

### C. Wild-Type Phenotype

Figure 1 illustrates a typical PDA protocol we used (filled and unfilled rectangles at the top) and the responses obtained with it from both mutant and wild-type flies. Although we used several variations of this protocol, they all consisted of a series of two or three bright blue stimuli followed by two or three bright orange stimuli. In the protocol illustrated in Figure 1, three blue stimuli of 4 s duration (Figure 1A, filled rectangles) were followed by three orange stimuli (unfilled rectangles) also of 4 s, all at 20-s interval. Since rhodopsin (Rh1) and metarhodopsin (M^*^) in R1–6 photoreceptors absorb maximally at ∼485 and ∼575 nm, respectively (see Section IA and Figure 3, bottom left), the photoequilibrium created by the blue stimulus would strongly favor photoconversion of Rh1 to M^*^, whereas the photoequilibrium created by the orange stimulus would strongly favor the reconversion of M^*^ to Rh1 in R1–6 photoreceptors. Thus, the blue stimuli would generate the PDA and the orange stimuli would terminate it. Before starting the stimulus series, typically the flies were first exposed to the orange stimulus to photoconvert any M^*^ that might have been present to Rh1, and dark adapted for a few minutes. Because the minority class of photoreceptors, R7/8, have different visual pigments with different absorption spectra (see Section IIA1 for references; reviews: Wang & Montell, 2007; Hardie, 2011), the same stimulus protocol does not generate the PDA in these photoreceptors. Thus, this protocol would not detect PDA defects specific for R7/8 photoreceptors.

The ERG responses obtained from wild-type flies (marked with *w*^−^) using the above protocol are shown in Figure 1A. The first blue stimulus generates a large response that lasts the duration of the stimulus (light-coincident component; Figure 1A, unfilled arrowhead), and after the stimulus is turned off, the response decays to a new level and persists at this new level in the dark. This is the depolarizing afterpotential (PDA) (Figure 1A, arrows). If left alone, it persists for tens of minutes in the dark. A fully developed PDA saturates the depolarizing capacity of the R1–6 photoreceptors, rendering them unresponsive to another stimulus. They are said to be “inactivated.”^1^ Thus, the second and third blue stimuli generate only small responses superposed on the PDA in extracellular ERG recording (Figure 1A, filled arrowheads). These small responses as well as a comparable portion of the response to the first blue stimulus do not come from R1–6 photoreceptors but from the R7/8 photoreceptors (Minke et al., 1975), in which the PDA is not generated by the stimulus protocol used. In fact, in transgenic flies in which phototransduction is blocked in R7/8 cells but not in R1–6 cells, a similar stimulus protocol would generate ERGs with these superposed responses and the corresponding portion of the first response missing (Figure 1, inset; Pearn et al., 1996). The first orange stimulus, which rephotoconverts M^*^ back to Rh, terminates the PDA and the ERG response returns to the baseline level, i.e., the R1–6 photoreceptors repolarize to the prestimulus level. Because of the broad absorption spectrum of Rh1, the second and third orange stimuli generate responses by activating Rh1 rephotoconverted from M^*^.

### D. Mutant Phenotypes

PDA-defective mutants (or “PDA mutants”) were identified and isolated by looking for those with phenotypes that deviate from the wild-type phenotype. From early on, it was clear that these mutants fell into two broad classes with multiple complementation groups in each (Pak, 1979; Stephenson et al., 1983). We named these two classes, *nina* (*neither inactivation nor afterpotential*) and *ina* (*inactivation but no afterpotential*) on the basis of their PDA phenotype, with a capital letter following the class designation (*nina* or *ina*) indicating the complementation group.

As the name implies, both classes of mutants are characterized by the absence or underdevelopment of the afterpotential, i.e., the PDA. This is particularly striking in strong *nina* mutants such as *ninaD^P245^* or *ninaE^P332^* (Figure 1B, C, arrows). As may be seen, there is no evidence of afterpotentials following the responses to blue stimuli in these mutants. Moreover, unlike in wild type, the second and third blue stimuli generate responses as big or nearly as big as that evoked by the first blue stimulus (Figure 1B, C, filled arrowheads). Previously, we explained the small responses obtained by the second and third blue stimuli in wild type, consisting exclusively of R7/8 cell responses, to the saturation of depolarizing capacity of R1–6 cells due to the fully developed depolarizing after-potential induced by the first blue stimulus. Since there is no afterpotential in *ninaE^P332^* or *ninaD^P245^*, the R1–6 cells are not saturated and can respond to the second and third blue stimuli with their full capacity. All *nina* mutants examined to date have been found to be reduced in Rh1 rhodopsin in varying degrees (Stephenson et al., 1983). As described earlier, the PDA is generated because of the inability of Arr2 to inactivate M^*^, which exists in a 5-fold molar excess of Arr2 (Dolph et al., 1993). In *nina* mutants with extremely small amounts of Rh1 (say, ≤ 1%), any M^*^ photoconverted from this small pool of Rh1 can be readily inactivated by the available Arr2 (*ninaE*^P332^ or *ninaD*^P245^ in Figure 1B and C). Hence, there is no afterpotential following the first blue stimulus and the second and third blue stimuli elicit full potentials (no inactivation). In the *nina* mutants with somewhat larger concentrations of Rh1, such as *ninaA*^P228^ with ∼ 15% Rh1, the afterpotential begins to develop following the cessation of the first blue stimulus but completely decays to the baseline during the interstimulus interval (not shown). In mutants with still larger amounts of Rh1, such as *ninaC*^P238^ with ∼35% Rh1, a PDA lasting the entire interstimulus interval develops, but it is much smaller than the fully developed PDA in wild type (Figure 1D, arrows). The second and third blue stimuli generate responses that are distinctly larger than those of wild type, though distinctly smaller than the response to the first blue stimulus (Figure 1D, filled arrowheads). Thus, these mutants show partial PDAs in parallel with partial inactivation. *ninaC* mutants also exhibit response waveforms different from those of *ninaD* or *ninaE* (Figure 1D), indicating that depletion of Rh1 may not be the only phenotype associated with *ninaC* mutants.

As with *nina's*, the mutants of the other class, *ina*, also fail to generate a sustained afterpotential (Figure 1E and F). Any afterpotential generated by the first blue stimulus decays within a few tens of seconds, the time course depending on the mutant, although the decay (inactivation) kinetics can be complex in some mutants (see Figure 1F). However, unlike in *nina's*, the subsequent blue stimuli elicit only small responses, presumably originating from R7/8 cells, even though the afterpotential is small or has even decayed completely to baseline (Figure 1E, filled arrowheads). Thus, although R1–6 cells are inactivated, unlike in *nina*, the inactivation does not depend on the level of depolarization induced in R1–6 cells by the previous blue stimuli. That is, inactivation of R1–6 photoreceptors is not due to saturation of the depolarizing capacity of these cells caused by the afterpotential generated by the previous blue stimulus, since there is little or no afterpotential.

**Figure 2:**
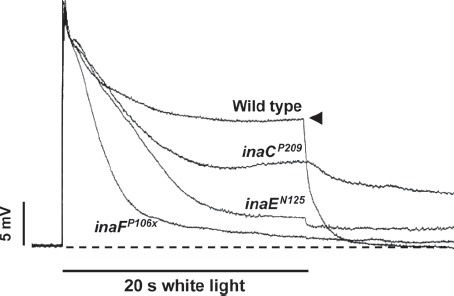
Intracellularly recorded photoreceptor responses of *ina* mutants and wild type. Photoreceptor responses to 20-s white stimuli recorded intracellularly from flies of the indicated genotypes are presented superposed on each other. In wild type, the response rapidly declines to a steady-state level (*filled arrowhead*), which depends on the intensity of the light stimulus, and remains there for the rest of the stimulus. This is a well-known adaptation response of photoreceptor to the light. In the *ina* mutants, on the other hand, the decline in amplitude (inactivation) that began at the onset of stimulus continues throughout the stimulus with the time course and the extent of decay depending on the mutant. The kinetics of decay can be complex in some mutants (*inaC*). Thus, the *ina* photoreceptor responses tend to terminate prematurely. The premature termination phenotype of a strong *ina* mutant, such as *inaF^P106x^*, closely resembles that of null *trp* (*transient receptor potential*) mutants.

The origin of inactivation in *ina* mutants is not well understood but appears to be associated with premature termination of the response that begins almost as soon as the stimulus is turned on. This phenomenon can be seen best in the responses elicited by stimuli of long durations. Shown in Figure 2 are intracellularly recorded responses of several *ina* mutants (*inaC^P209^*, *inaE^N125^*, *inaF^P106x^*) and wild type to 20-s white stimulus superposed on each other. In wild type, after the initial peak, the response decays to the steady-state level (Figure 2, filled arrowhead) and remains there for the duration of stimulus, whereas in *ina's* the response steadily declines during the entire duration of stimulus, the time course and extent of decay depending on the mutant. The premature decay of response during a stimulus is a well-known defining characteristic of the mutants of the phototransduction channel gene *trp* (Cosens & Manning, 1969; Minke et al., 1975), although in general, the response decay in *trp* is much faster than in most *ina* mutants (see also Leung et al., 2008, Figure 1A). Another important phenotype of *trp* mutants is the pronounced refractory period, which prevents generation of full-amplitude response for a minute or more following an initial response to a bright stimulus (Leung et al., 2008). The *ina* mutants that have been examined also display similar refractory periods, although the duration is not as long and the degree of suppression of subsequent responses is not as severe as in *trp* mutants (Leung et al., 2008). In these two important aspects, the *ina* mutants appear to represent milder forms of *trp* mutants. Thus, it appears as though in these mutants the response generating mechanism itself steadily inactivates during stimulus and remains inactivated for a time after the stimulus. In the case of *trp*, Hardie et al. (2001) presented evidence that these defects are attributable to depletion of PIP_2_ (phosphatidylinositol 4,5-bisphosphate), the substrate for the key effector enzyme for phototransduction, PLC_β_ (phospholipase C_β_). There are two classes of phototransduction channels in *Drosophila* photoreceptors: highly Ca^2+^-permeable TRP and nonselectively cation-specific TRPL (Hardie & Minke, 1992). In *trp* mutants, the only light response remaining is that mediated through the TRPL channels. This response rapidly decays because, in the absence of sufficient Ca^2+^ influx through the TRP channel to inhibit PLC_β_ activity, PIP_2_ is depleted rapidly (Hardie et al., 2001). The refractory period presumably represents the time required for regeneration of PIP_2_. It is not known whether the same or similar mechanisms are responsible for the *ina* phenotype. Nevertheless, the phenotype suggests that *ina* mutations affect some fundamental properties of phototransduction, particularly in relation to TRP channel functions.

### E. List of PDA Mutants

Table 1 lists the *nina* and *ina* mutants in our collection and the genes identified by them. Most of these mutants were isolated by ethyl methanesulfonate (EMS) mutagenesis in the 6-year period between 1975 and 1981 (see review: Pak, 2010), although it took much longer to sort out their complementation groups. Most of them are on the autosomes because they were isolated using autosome-specific mutagenesis schemes (see Pak, 1979). In addition to EMS mutagenesis, Joe O'Tousa and Quentin Pye carried out hybrid dysgenic crosses^2^ in 1982 to isolate alleles of *ninaA*, *ninaB*, *ninaE*, and *ninaG*. These are indicated by a small “d” following the isolate (allele) number in the superscript in Table 1. Gregore Koliantz also used this method in 1987–1988 to isolate *nina* mutants defining two new complementation groups, *ninaJ* and *ninaI*, mapping to the chromosomes X and II, respectively. The first *inaF* mutant, *inaF^P105p^*, was generated by *Δ2–3*-mediated P-element mutagenesis in 1997 by Chenjian Li and Lydia Strong (Li et al., 1999). Additional alleles were generated in this gene by mobilizing the above allele to induce imprecise excisions.

**Table 1:** nina and ina mutant alleles in the Pak laboratory.

Mutant	Gene function	CG no.	Locus	Isolated in the Pak laboratory	Isolated in other laboratories
*ninaA*	Cyclophilin A	CG3966	2L:21E2	*P228, P263, P268, P271d, P273d*	
*ninaB*	β-Carotene oxygenase	CG9347	3R:87F11	*P315, P319, P360d*	
*ninaC*	Myosin III	CG5125	2L:27F3	*P216, P221, P225, P230, P235, P238, P239, P240, P257, P262, P266*	*US2985* (M)
*ninaD*	Scavenger receptor	CG31783	2L:36E3	*P245, P246, P258, P261*	
*ninaE*	Rh1 opsin	CG4550	3R:92B4	*P318, P322, P332, P334, P344, P350, P352, P353, P354d, P361, P362*	*RH27* (M), *RH88* (M), *US6275* (M), *ora*JK84 (M), *Eng* (E)
*ninaF*			3R:84F14;85A3	*P342*	
*ninaG*	GMC oxidoreductase	CG6728	3R:86E5	*P330, P355d, P356d, P357d, P358d, P359d*	
*ninaH*			3R:93B6;C6	*P314, P351*	
*ninaI*			X	*P82d, …, P93d, P100d*	
*ninaJ*			2	*P272d, P274d, …, P283d*	
*inaA*			2R:49A1; B3	*P226*	
*inaB*			2L:25D6;E1	*P222, P223*	*JK1669* (M)
*inaC*	Eye PKC	CG6518	2R:53E1	*P207, P209, P234*	*US2167* (M)
*inaD*	Scaffold protein	CG3504	2R:59B3	*P215*	*US6545* (M)
*inaE*	DAG lipase	CG33174	X:12C4;C5	*P19*	*N125* (H)
*inaF*	TRP expression/regulation	CG42447	X:10D8;E1	*P105p, P106x, P111x*	

*Note.* M = isolated in the John Merriam laboratory; H = isolated in the Martin Heisenberg laboratory; E = recovered from stocks sent by William Engels.

Some PDA mutants have altered ERG waveforms that can be detected in conventional ERG recordings without using the PDA protocol. Thus, several of our mutants in Table 1 were actually isolated before the PDA protocol for mutant screening was formally put in place in September 1975. They are *inaC^P207^* (1/74), *inaC^P209^* (3/74), *inaD^P215^* (8/74), *inaE^P19^* (5/69), and *ninaC^P216^* (8/74), with dates of isolation in parentheses. Their PDA phenotype was not known at the time of isolation, and their identity as PDA mutants became known years later through PDA recordings and complementation tests with known PDA mutants.

Table 1 also lists several mutants isolated in other laboratories, mostly in the John Merriam laboratory at UCLA (University of California–Los Angeles). These are listed in a separate column for each gene. The dates of isolation are not known. The dates we acquired them are also obscure, although we most likely obtained most of them in the early 1980s. As in the case of the previous mutants, they were isolated without using the PDA protocol and were assigned to the respective complementation groups on the basis of genetic complementation tests. In the case of *inaE^N125^*, obtained from Martin Heisenberg in Germany, the isolation was based on behavioral optomotor assays (Heisenberg, 1971). In all these cases, their identification as PDA mutants came years later. For example, for *inaD^US6545^*, which is homozygous lethal possibly due to a second-site mutation, its assignment to the *inaD* complementation group did not come until July 1995.

## II. CONTRIBUTIONS OF *nina/ina* MUTANTS TO PHOTORECEPTOR FUNCTION

### A. The *nina* Mutants

We will now discuss how *nina*/*ina* mutants have contributed to our knowledge of photoreceptor function. We will start with the *nina* mutants. The cardinal feature of all *nina* mutants is an allele-dependent depletion of the visual pigment Rh1 in R1–6 photoreceptors^3^ (Stephenson et al., 1983). In *Drosophila*, impairments of synthesis of either the protein (opsin) or the chromophore moiety of rhodopsin can lead to rhodopsin depletion. These two mechanisms appear to account for the phenotypes of the majority of *nina* mutants. Another important mechanism appears to be impairment in maturation and intracellular trafficking of nascent opsin, thereby resulting in depletion of rhodopsin. There are also *nina* mutants, such as *ninaC*, in which depletion of Rh1 appears to be only one of the many phenotypes and may not even be their major one.

#### 1. *ninaE*

The *ninaE* gene encodes the major class of opsin, Rh1, expressed in R1–6 photoreceptors (O'Tousa et al., 1985; Zuker et al., 1985). As the gene encoding the first molecule in the phototransduction cascade, this gene was the first to be targeted for molecular cloning. As the number of mutants displaying the *nina* phenotype accumulated and the complementation groups into which they fell became identified, we came to realize that one common feature all *nina* mutants seemed to share was Rh1 rhodopsin depletion (Stephenson et al., 1983). We hypothesized that one of the five *nina* complementation groups (*ninaA*, *B*, *C*, *D*, and *E*) we had already identified at the time might actually correspond to the Rh1 opsin gene. To test this hypothesis, the available *nina* mutations were tested for their R1–6 cell-line specificity under the assumption that mutations in any gene encoding R1–6 opsin would only affect R1–6 opsin, and not the minor opsins in R7 or R8. Mutations in two genes, *ninaA* and *ninaE*, satisfied this requirement (Larrivee et al., 1981; Schinz et al., 1982; Stephenson et al., 1983). In genetic tests for gene-dosage effect, the *ninaE* gene (Scavarda et al., 1983), but not the *ninaA* gene (Pak et al., 1980), was found to affect the Rh1 rhodopsin content in a dosage-dependent manner, making it very likely that it is the Rh1 opsin gene. This information was crucial for the cloning of this gene.

The *ninaE* gene was cloned by homology to bovine opsin (O'Tousa et al., 1985; Zuker et al., 1985). It was the first invertebrate opsin gene to be cloned. At the time, complete sequences were known for only two other opsins, bovine and human (Nathans & Hogness, 1983, 1984). These were also the first G protein–coupled receptors to have their complete sequences determined. Although the overall amino acid identity between NINAE and bovine opsin was fairly modest (∼36%), NINAE displayed key structural features of opsin. Most notably, it was found to have seven predicted transmembrane segments, with the 7th transmembrane segment containing a conserved lysine residue that would serve as the chromophore linkage site. As with mammalian opsins, there were also several potential phosphorylation sites near the C-terminus.

Cloning of *ninaE* allowed all five minor classes of opsin to be cloned and their sequences and cellular expressions determined: Rh2 in ocelli (Cowman et al., 1986; Mismer et al., 1988; Pollock & Benzer, 1988), Rh3 and Rh4 in R7 photoreceptors (Zuker et al., 1987; Montell et al., 1987; Fryxell & Meyerowitz, 1987), and Rh5 and Rh6 in R8 photoreceptors (Chou et al., 1996; Papatsenko et al., 1997; Salcedo et al., 1999). A major function of R7 and R8 photoreceptors is color perception. R7 and R8 have shorter rhabdoremeres than R1–6. They are located in the middle of the ommatidium and span only one half of the retina with the R7 rhabdomere located on top of the R8 rhabdomeres. R7 photoreceptors express stochastically either Rh 3 or Rh4 opsins (see above for references), both of which are ultraviolet (UV)-absorbing, in a 30/70 ratio. Expression of Rh3 in R7 is coupled with expression of Rh5 (blue) in R8, whereas expression of Rh4 in R7 is coupled with that of Rh6 (green) in R8 (Chou et al., 1996; Papatsenko et al., 1997; Chou et al., 1999). This pattern of expression defines two spectrally distinct classes of R7/8 photoreceptors, expressing either Rh3/Rh5 or Rh4/Rh6 pair, distributed randomly throughout the eye in a 30/70 ratio. Molecular details of how this coordinate expression of minor opsins in R7 and R8 photoreceptors is achieved have now been largely worked out (reviews: Morante et al., 2007; Jukam & Desplan, 2010).

It is now widely recognized that mutations in the opsin gene cause degeneration of photoreceptors in both *Drosophila* and humans. The first indication that mutation in an opsin gene might cause degeneration of photoreceptors came from observations on the *Drosophila* mutant *ora^JK84^* (Koenig, 1975; Koenig & Merriam, 1977). It was first reported that *ora^JK84^* mutants are almost completely devoid of rhabdomeres in R1–6 photoreceptors, but not in R7/8 photoreceptors (Koenig, 1975; Harris et al., 1976). Subsequently, it was found that irregular, loosely packed rhabdomeres do form but quickly degenerate by 1 week post eclosion (Stark & Sapp, 1987; O'Tousa et al., 1989). O'Tousa et al. (1989) showed (1) that *ora*^JK84^ is a double mutant carrying a mutation in the *ninaE* gene and another mutation in the nearby *ort* gene and (2) that the mutation in the *ninaE* gene is solely responsible for the mutant's degeneration phenotype. These findings on *Drosophila* presaged those then emerging from studies on humans. In humans, a candidate gene approach was used to establish a correlation between a mutation in a gene and retinitis pigmentosa (RP). The rhodopsin gene was the first gene to show such correlation (Dryja et al., 1990; Inglehearn et al., 1991; Sung et al., 1991). It has been estimated approximately 10% of all cases (or approximately 20–25% of autosomal cases) of RP and allied retinal degenerations are due to mutations in the rhodopsin gene (Dryja & Berson, 1995).

Some mechanistic understanding has been achieved for some forms of *ninaE*-mediated degeneration. Retinal degeneration in *ninaE* null mutants, which include *ora*^JK84^, is distinct from those in hypomorphs and appears to be due to rhodopsin being required in rhabdomere morphogenesis (Kumar & Ready, 1995). Unlike in hypomorphs, rhabdomere defects in *ninaE* nulls begin during rhabdomere morphogenesis. As a consequence, structurally normal rhabdomeres never form, and developing rhabdomere membrane, instead of turning into rhabdomeres, catastrophically involutes into the cell as “curtains of apposed membrane.” Even very small amounts of rhodopsin appear to be sufficient to rescue mutants from the above fate (Johnson & Pak, 1986; Leonard et al., 1992). These hypomorphic *ninaE* mutants produce smaller than normal rhabdomeres containing structurally normal microvilli, which degenerate over a period of weeks after eclosion (Leonard et al., 1992).

Many *ninaE* mutations are dominant, as are also many mammalian rhodopsin mutations. Two groups developed genetic screens to isolate dominant *ninaE* mutants (Colley et al., 1995; Kurada & O'Tousa, 1995). Some of these mutants carry identical amino acid substitutions as in human autosomal dominant RP (ADRP) patients. In the case of mammals, one of the common mechanisms of dominant rhodopsin-mediated degeneration was found to be faulty maturation and cellular trafficking of nascent rhodopsin (Roof et al., 1994; Sung et al., 1994). The dominant *ninaE* mutants were used to address the above question as well as to determine why the dominant *ninaE* heterozygotes with 50% wild-type rhodopsin molecules also degenerate (Colley et al., 1995; Kurada & O'Tousa, 1995; Kurada et al., 1998). They found that the mutant proteins interfere with the maturation of the wild-type proteins. These proteins (mutant as well as immature wild-type) do not reach the rhabdomeres and accumulate in the endoplasmic reticulum (ER), causing proliferation of ER cisternae and ultimately degeneration (Colley et al., 1995).

Another mechanism of dominant rhodopsin-mediated degeneration in mammals was found to be constitutive activity of rhodopsin (Robinson et al., 1992; Dryja et al., 1993; Rao et al., 1994). The constitutively active dominant *ninaE* allele, *NinaE*^pp100^, was isolated in a genetic screen (Iakhine et al., 2004). However, constitutive opening of light-sensitive channels seems to make only a minor contribution to degeneration in this mutant, since near-null mutations in the PLC_β_ gene(*norpA*), and the TRP channel gene (*trp*), suppress the degeneration phenotype only weakly. Instead, the major mechanisms of degeneration appear to be (1) the persistent formation the NINAE^pp100^-Arr2 complex and (2) the elevated levels of Gqα in the cytosol. Activation of rhodopsin to metarhodopsin causes (1) Arr2 to bind to metarhodopsin to inactivate the latter and (2) Gqα to translocate to the cytosol as a long-term mechanism of adaptation. If rhodopsin is constitutively active, both these events would occur persistently. Stable meta-Arr2 complexes have been shown to be endocy-tosed and to cause apoptosis of photoreceptors (Alloway et al., 2000; Kiselev et al., 2000). The persistent localization of Gqα in the cytosol appears to be a novel major cause of degeneration, independent of the Arr2-dependent mechanism.

Recently, a completely new function has been discovered for Rh1 rhodopsin, temperature sensing in *Drosophila* larvae (Shen et al., 2011). Generally, temperature sensing in animals is mediated through direct activation of transient receptor potential (TRP) cation channels (Caterina, 2007; Bandell et al., 2007). In *Drosophila* larvae, however, discrimination between 18°C temperature, which is optimal for their survival, and slightly higher temperatures (19–24 °C) is accomplished indirectly through a signal transduction cascade similar to that used in *Drosophila* phototransduction (Kwon et al., 2008). The receptor molecule initiating this cascade appears to be Rh1 rhodopsin, since mutations in the *ninaE* gene eliminate this thermosensory response (Shen et al., 2011).

#### 2. *ninaA*

Early on, the *ninaA* gene sparked interest because mutations in this gene, along with those in the *ninaE* gene, were found to reduce the rhodopsin content specifically in R1–6 photoreceptors (Larrivee et al., 1981; Stephenson et al., 1983). The molecular basis of the R1–6 cell-line specificity of *ninaE* was elucidated with the demonstration that *ninaE* encodes Rh1 opsin (apoprotein of rhodopsin) specifically expressed in R1–6 cells (previous section). However, the specificity of *ninaA* for R1–6 cells was not clear.

The *ninaA* gene was found to encode a 237-aa (amino acid), cyclophilin homolog (Schneuwly et al., 1989; Shieh et al., 1989). Because cyclophilins were shown to be peptidyl prolyl *cis-trans* isomerases (Takahashi et al., 1989; Fischer et al., 1989) implicated in catalyzing protein folding, it was first thought that the NINAA protein might be required for the proper folding and stability of Rh1. However, the following lines of evidence demonstrated that NINAA acts as a chaperone in intracellular trafficking of nascent rhodopsin from the endoplasmic reticulum (ER), where opsin is synthesized, to the rhabdomeres. NINAA is expressed in the ER and secretory vesicles, and, in *ninaA* mutants, transport of Rh1 out of the ER is strongly inhibited, leading to dramatic Rh1 retention in ER and accumulations of ER cisternae (Colley et al., 1991) and eventual degeneration of photoreceptors (Colley et al., 1995). The NINAA protein forms a stable and specific complex with Rh1, and maturation of Rh1 rhodopsin depends quantitatively on the amount of NINAA available (Baker et al., 1994). The specificity of *ninaA* mutations for Rh1 was shown to arise from the substrate specificity of NINAA for Rh1 opsin (Stamnes et al., 1991).

The NINAA protein was the first membrane-associated accessory protein identified to play an important role in the maturation of a G protein–coupled receptor (GPCR: rhodopsin, in this case) (Colley et al., 1991; review: Bermak & Zhou, 2001). In searching the bovine genome for NINAA-homologous genes highly expressed in the retina, Ferreira et al. (1995) identified the RanBP2 (Ran-binding protein 2) gene, encoding a large protein that binds the GTPase Ran (Yokoyama et al., 1995; Wu et al., 1995). Two contiguous domains of this multidomain protein, Ranbinding domain 4 (RBD4) and the adjacent cyclophilin domain, were shown to act in concert as a chaperone to facilitate the biogenesis of the red/green visual pigment in the dichromatic bovine retina (Ferreira et al., 1995). In *Caenorhabditis elegans*, ODR-4 (abnormal odorant response 4) was shown to be required for the proper targeting of the odorant receptor ODR-10 to olfactory cilia (Dwyer et al., 1998). ODR-4 is a 445-aa protein expressed exclusively in the olfactory organ with a similar structural topology but no obvious sequence similarity to NINAA. It may be that many GPCRs require specific chaperone/accessory proteins of their own for proper folding/maturation.

In vertebrates, export of GPCRs from the ER and transport to the Golgi and the cell surface has been extensively studied and found to be a highly regulated dynamic process requiring many players at several different stages of regulation (reviews: Bermak and Zhou, 2001; Dong et al., 2007). A crucial step in this regulation is interactions of GPCRs with chaperone proteins in the ER. Among the best characterized ER chaperone proteins for GPCRs are calnexin, calreticulin, and BiP (Kleizen & Braakman, 2004; Williams, 2006). In the *Drosophila* eye, the protein products of one or the three known calnexin genes, *cnx99A*, were found to be required as chaperones specifically for Rh1 maturation, much like NINAA (Rosenbaum et al., 2006). CNX99A also acts as a regulator of Ca^2+^ entering the photoreceptors during phototransduction. Mutations in this gene cause light-enhanced retinal degeneration.

#### 3. *ninaB, D, G, santa maria*, and *pinta:* Genes Required for Chromophore Synthesis

Animals depend on dietary intake of carotenoids and xan-thophills (mainly β-carotene) for the production of the chromophore. Although retinal (the aldehyde of vitamin A) is commonly used as the chromophore in most vertebrates, chemically related compounds are also utilized for this purpose in various species throughout the animal kingdom. For example, (3,4)-didehydroxyretinal is found in fish and amphibians (Suzuki et al., 1984) and 3-hydroxyretinal, derived from vitamin A3 (3-hydroxyretinol), is found in insects (Vogt, 1989). The two enantiomers of 3-hydroxyretinal appear to be differentially utilized in insects. Whereas the lower order dipterans use (3*R*)-3-hydroxyretinal, members of the higher *Diptera*, *Cyclorrapha*, which include *Drosophila*, use (3*S*)-3-hydroxyretinal (Seki et al., 1994), at least for the majority class of photoreceptors, R1–6 (Ahmad et al., 2006).

It was realized since the early 1980s that, in a substantial fraction of *nina* mutants, defects in chromophore synthesis rather than opsin synthesis was the basis of rhodopsin depletion (Stephenson et al., 1983). Thus, mutants in two of the five *nina* genes tested by these authors, *ninaB* and *D*, could be rescued by dietary supplementation with carotenoids or retinoids. Moreover, *ninaB* and *D* mutants exhibited different substrate preferences for rescue. Whereas *ninaD* mutants could be rescued by a broad range of carotenoids and retinoids, *ninaB* seemed to show a preference for retinal, suggesting that the *ninaB* gene product may act downstream of the *ninaD* product. Since that time, three other genes that function in chromophore synthesis have been identified, *santa maria* (*scavenger receptor acting in neural tissue and majority of rhodopsin is absent*) (Wang et al., 2007), *pinta* (*PDA is not apparent*) (Wang & Montell, 2005), and *ninaG* (Sarfare et al., 2005; Ahmad et al., 2006). The *santa maria* and *pinta* mutants were isolated by the Craig Montell group at Johns Hopkins using a mutant selection scheme similar to the “PDA protocol” we have described. The first *ninaG* mutant, *ninaG*^P330^, was isolated in 1979, as part of the original PDA-based screen. The remaining alleles were isolated in hybrid dysgenesis screen in 1982 by Joe O'Tousa and Quentin Pye, both of whom were in the Pak laboratory at the time. Studies of mutants in these five genes form the basis of our current understanding of the chromophore synthetic pathway in *Drosophila*.

The first of the chromophore-related genes to be characterized was *ninaB*. It encodes a β,β′-carotene-15,15′-monooxygenase (BCO), which was shown in an *in vitro* assay to symmetrically cleave β-carotene at the 15-15' double bond to yield two molecules of retinaldehyde (von Lintig & Vogt, 2000). The NINAB protein was the first invertebrate member of the β-carotene cleavage oxygenase I (BCO1) family to be characterized. In living flies, however, dietary β-carotene is reported to be preferentially hydroxylated to form zeaxanthin (3,3′-dihydroxy β,β′-carotene) (Giovannucci & Stephenson, 1999; Voolstra et al., 2006). Detailed enzymatic analyses of both moth and *Drosophila* NINAB suggest that NINAB can use zeaxanthin as a substrate to yield two molecules of 3-dihydroxyretinaldhyde (Oberhauser et al., 2008; Voolstra et al., 2010). This would occur outside the retina, since NINAB is expressed in extraretinal neurons and glia in the head (Wang et al., 2007).

NINAB has been reported to function also as an isomerase. NINAB, like other the members of the carotenoid cleavage oxygenase family, shows significant sequence homology to mammalian RPE65 (retinal pigment epithelium 65-kDa protein), which is important in the reisomerization of all-*trans* retinol in mammals (Jin et al., 2005). Indeed, a NINAB homolog in the moth *Galleria mellonella* was found to exhibit both oxygenase and isomerase activities in that it catalyzes the conversion of zeaxanthin (3,3′-dihydroxy β,β′-carotene) into equal parts of all-*trans* and 11-*cis* dihydroxy retinaldehydes (Oberhauser et al., 2008). The combined oxygenase and isomerase activities of NINAB may also be the basis of the earlier observation that *Drosophila* can biosynthesize 11-*cis* 3-hydroxyretinal from carotenoid in the dark (Seki et al., 1986; Isono et al., 1988).

**Figure 3:**
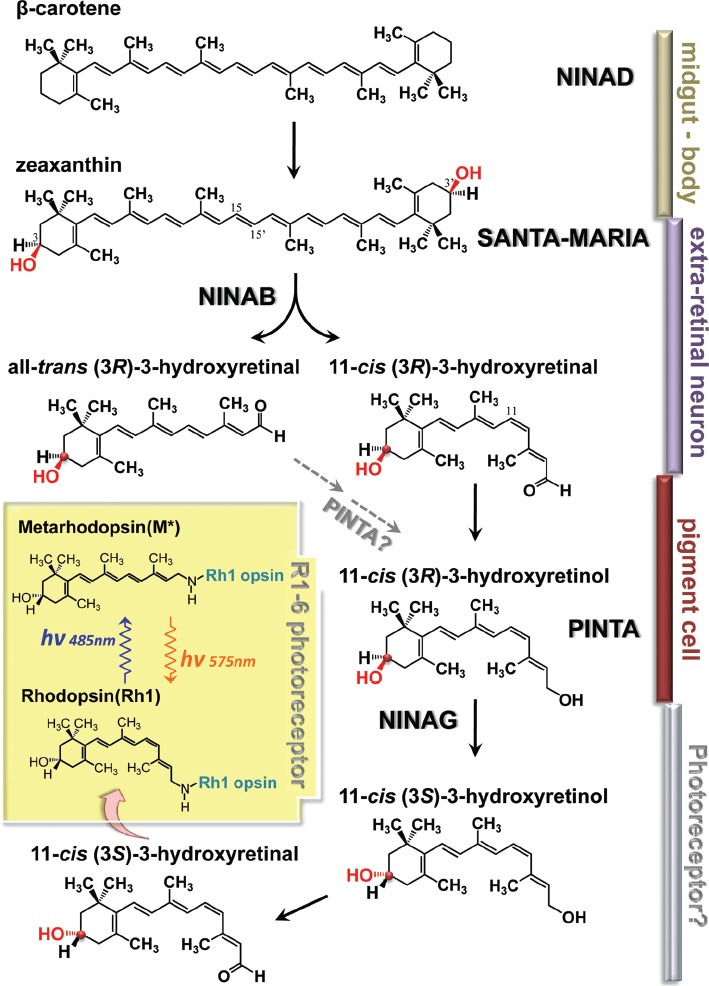
Schematics of putative chromophore biosynthetic pathway. Dietary carotenoids and xanthophils are taken up in the midgut by NINAD and carried by circulation to extra retinal neurons and glia to be taken up there by SANTA MARIA. The dietary β-carotene, once taken up, is reported to be rapidly hydroxylated to form zeaxanthin by unknown mechanisms. In extraretinal neurons and glia, NINAB cleaves and isomerizes zeaxanthin to yield 11-*cis* (3*R*)-3-hydroxyretinal and all-*trans* (3*R*)-3-hydroxyretinal. Presumably, the chromophore is generated from both products. 11-*cis* (3*R*)-3-hydroxyretinal (right branch in Figure 3) is converted to its retinol form, 11-*cis* (3*R*)-3-hydroxyretinol by a still uncharacterized retinal dehydrogenase. In the mean time, 11-*cis* (3*R*)-3-hydroxyretinol may also be generated from all-*trans* (3*R*)-3-hydroxyretinal (left branch in Figure 3) by conversion to its retinol form and 11-*cis* isomer. PINTA preferentially binds the retinol form of the retinoids in pigment cells and this binding is required for the chromophore biogenesis, though its precise role is not yet clear. In the final step of chromophore synthesis, 11-*cis* (3*R*)-3-hydroxyretinol is converted to its 3*S* stereoisomer by NINAG to yield 11-*cis* (3*S*)-3-hydroxyretinol. This isomerization takes place about the chiral center at the C-3 position of the retinoid ring. C-3 is marked by the red dot and the highlighted OH residue. 11-*cis* (3*R*)-3-hydroxyretinol is converted to 11-*cis* (3*R*)-3-hydroxyretinal to bind opsin (NINAE) expressed in R1–6 photoreceptors by forming Schiff base linkage with the lysine residue. Inset depicts photo-interconversion of rhodopsin (Rh1) and metarhodopsin (M^*^).

Two genes that encode proteins likely to be involved in the uptake of dietary β-carotene have been identified, *ninaD* and *santa maria* (Kiefer et al., 2002; Wang et al., 2007). Kiefer et al. (2002) showed that *ninaD* encodes a *Drosophila* homolog of class B scavenger receptor type 1 (SRB1). Genes of the SRB1 family are conserved throughout metazoans and encode proteins implicated in lipid binding and transport, particularly cholesterol transport in mammals (Acton et al., 1996). It was therefore suggested NINAD likely plays a role in the uptake of β-carotene. However, since NINAD is expressed in the midgut, whereas NINAB is expressed in extraretinal neurons and glia in the head (Wang et al., 2007), it seemed unlikely that carotenoids taken up in the midgut via NINAD is the substrate of NINAB for centric cleavage. Wang et al. (2007) isolated a mutant in a new locus, *santa maria*, encoding another SRB1. In contrast to NINAD, SANTA MARIA is co-expressed with NINAB in glia and extraretinal neurons. Thus, the picture that emerges is that dietary β-carotene is taken up in the midgut via NINAD and carried by circulation to glia and extraretinal neurons to be taken up there via SANTA MARIA, probably as zeaxanthin, the hydroxylated form, and NINAB uses it as substrate for cleavage (Figure 3).

The protein products of two other genes have been proposed to participate in the subsequent steps of chromophore synthesis, *pinta* (Wang & Montell, 2005) and *ninaG* (Sarfare et al., 2005; Ahmad et al., 2006) (see Figure 3). The *pinta* gene encodes a retinoid-binding protein, with preference for all-*trans* retinol, expressed and required in the retinal pigment cells for the production of the chromophore. This finding was the first to implicate the retinal pigment cells in the production of the chromophore in *Drosophila*. However, the precise cellular function of PINTA has not yet been elucidated. Two possibilities, which are not mutually exclusive, have been proposed (Wang & Montell, 2005): (1) it sequesters all-*trans* retinol in pigment cells thereby generating a concentration gradient that favors uptake of retinol, and/or (2) it facilitates the presentation of all-*trans* retinols to the enzymes involved in the next step of chromophore synthesis. In light of the previous discussion, however, the retinoids PINTA binds may actually be 3-hydroxyretinoids rather than retinoids (without the hydroxyl residue). PINTA binding of 3-hydroxyretinoids was not investigated by these authors.

The *ninaG* gene encodes a glucose-methanol-choline (GMC) oxidoreductase proposed to act in the conversion of (3*R*)-3-hydroxyretinol to the 3 *S* enantiomer in the retina (Figure 3; Ahmad et al., 2006). The conversion of 3*R* to 3*S* form occurs through isomerization about the chiral center at the C3 position of the retinoid ring (The C3 position is marked with a red dot in Figure 3). In *ninaG* mutants, synthesis of the chromophore (3*S*)-3-hydroxyretinal is blocked (Sarfare et al., 2005). Moreover, in transgenic flies ectopically expressing Rh4 visual pigment in the majority class photoreceptors, R1–6, when placed on a *ninaG* mutant background, large amounts of 11-*cis*3-hydroxyretinol accumulate, suggesting that 11-*cis*3-hydroxyretinol is an intermediate just upstream of the *ninaG* block. They thus proposed that the accumulating intermediate is in the 3*R* form and NINAG acts in the final step of chromophore production, the conversion of (3*R*)-3-hydroxyretinol to the 3*S* enantiomer (Sarfare et al., 2005; Ahmad et al., 2006) (Figure 3). In the *Drosophila* eye, only Rh1 rhodopsin expressed in R1–6 photoreceptors use (3*S*)-3-hydroxyretinal as its chromophore, and the rhodopsins expressed in the minor class of photoreceptors, such as Rh4, have (3*R*)-3-hydroxyretinal–based chromophore. Thus, only Rh1 rhodopsin is depleted in *ninaG* mutants. In the experiment mentioned above, the authors used transgenic flies ectopically expressing Rh4 in R1–6 photoreceptors to avoid the possibility that any alterations in the amount of retinoids that they observe might be secondary effects of rhodopsin depletion.

#### 4. *ninaC*

The *ninaC* gene encodes a class III myosin expressed predominantly in the eye (Montell & Rubin, 1988). Myosin III differs from all other classes of myosins in having an N-terminal kinase domain. The two *Drosophila* NINAC isoforms were the first class III myosins to be identified. Since then, a NINAC homolog has been identified in the *Limulus* eye (Battelle et al., 2001), in striped sea bass (Dosé et al., 2003), and humans (Dosé and Burnside, 2000; Berg et al., 2001). Human myosin III and *Drosophila* NINAC have been shown to bind actin in an ATP-dependent manner (Hicks et al., 1996; Doséet al., 2003), and human myosin III has been shown to perform plus-end–directed motor activity (Komaba et al., 2003; Kambara et al., 2006). One of the two human MyoIII isoforms, MyoIIIA, is highly expressed in the retina and cochlea and mutation in MyoIIIA gene has been reported to cause non-syndromic hearing loss (Walsh et al., 2002). However, little is known about cellular functions of vertebrate MyoIII, and to date most of the details of *in vivo* functions of MyoIII have come from the work on the *Drosophila* eye. Unlike human myosin III, motor function has not yet been directly demonstrated for NINAC.

The *ninaC* gene encodes two splice variants: a 174-kDa protein (p174) localized to the rhabdomeres and a 132-kDa protein (p132) localized to the cell body (Montell & Rubin, 1988; Porter et al., 1992). The NINAC protein is a multifunctional protein, and strong *ninaC* mutants exhibit a wide range of phenotypes. These include reduced visual pigment contents (Stephenson et al., 1983), ultrastructural microvillar abnormality (Matsumoto et al., 1987; Hicks & Williams, 1992), defects in response termination and adaptation (Porter et al., 1992, 1993), loss of rhabdomeral calmodulin (Porter et al., 1993), and light-dependent retinal degeneration (Porter et al., 1992), etc. For many of these phenotypes, it is not yet clear what their molecular bases are or how they are related to each other. We will discuss some of these below.

Reduction in visual pigment content was one of the first phenotypes to be noted in this mutant (Stephenson et al., 1983). Also noted early was the loss of the central axial cores of the rhabdomeral microvilli in strong *ninaC* mutants in electron microscopy (Matsumoto et al., 1987). The axial cytoskeleton, consisting of actin filaments, appears as a single structure with side arm linkages to the microvillar plasma membrane in electron microscopy (EM) (Arikawa et al., 1990). Hicks et al. (1996) showed that *ninaC* mutants lacking the p174 isoform display a fragmented axial structure without side arm linkage even before eclosion, eventually resulting in degeneration of rhabdomeres. They suggested that p174 may have a role in forming the structural linkage between the axial cytoskeleton and the microvillar membrane, in a manner similar to that of brush border myosin I in the intestinal microvilli (Matsudaira & Burgess, 1979). This hypothesis, if correct, could provide common explanations for some of the *ninaC* phenotypes.

One of the salient characteristics of the ERG of strong *ninaC* mutants is the slow response termination following light off (slow deactivation—see footnote 1) (Porter et al., 1992, 1993). Deactivation of the light response is at least in part Ca^2+^-regulated and appears to be mediated through the Ca^2+^-binding regulatory protein, calmodulin (CaM). In *Drosophila* photoreceptors, CaM is highly enriched in the rhabdomeres (Porter et al., 1993). The rhabdomeral localization of CaM is determined by the NINAC isoform p174, which localizes specifically to rhabdomeres itself. Thus, the CaM localization to the rhabdomeres is severely reduced in mutants that do not express the p174 isoform or in which CaM binding sites in p174 are eliminated (Porter et al., 1993, 1995). Moreover, the elimination of CaM sites results in slow termination of light response (ibid.). Similarly, hypomorphic mutants of the CaM gene, *cam*, display slow prolonged response termination (Scott et al., 1997).

The NINAC protein has also been implicated in the light-dependent shuttling of signaling proteins into and out of the rhabdomeres during long-term adaptation. Arrestin 2 (Arr2) (Kiselev et al., 2000; Satoh & Ready, 2005), the α subunit of G protein (Gqα) (Kosloff et al., 2003; Cronin et al., 2004), and the TRPL channel (Bähner et al., 2002) have all been shown to translocate in a light-dependent manner to regulate the sensitivity of the phototransduction machinery during light and dark illumination conditions. Thus, Gqα and TRPL, both of which contribute to activation of phototransduction, shuttle out of the rhabdomeres during illumination and translocate back into the rhabdomeres in the dark, whereas Arr2, which inactivates phototransduction, does the opposite. Lee et al. (2003) reported that translocation of Arr2 into the rhabdomeres requires phosphoinositide (PI)-mediated interactions of Arr2 with NINAC. However, this finding was disputed by another group, which found no evidence of NINAC-dependent shuttling of either Arr1 or Arr2 (Satoh & Ready, 2005). Translocation of Gqα into the rhabdomeres has also been reported to require NINAC (Cronin et al., 2004). In both these cases, NINAC is required for shuttling into the rhabdomeres. Assuming that NINAC is a plus-end–directed motor, as the human MyoIII was shown to be (Komaba et al., 2003; Kambara et al., 2006), this direction of movement is consistent with the polarity of actin filaments, which are arranged with the plus ends toward the distal end of the microvilli (Arikawa et al., 1990). However, in the case of TRPL shuttling, *ninaC* null mutation is reported to disrupt movements out of the rhabdomeres (Meyer et al., 2006). Unlike the other two signaling proteins, TRPL is a membrane protein. It is possible that TRPL does not utilize the NINAC-based movement. The above authors suggested that the observed disruption of translocation in *ninaC* may be a secondary effect of degeneration in *ninaC*.

NINAC is also involved in Ca^2+^-dependent inactivation of metarhodopsin (M^*^) (Liu et al., 2008). Following photoconversion of rhodopsin to M^*^, M^*^ is inactivated by Arr2 binding. Liu et al. (2008) measured the lifetime of M^*^ using a two-flash technique in which the first flash photoactivates rhodopsin and the second flash activates M^*^. If the second flash hits M^*^ before it is inactivated by Arr2, photoconversion of M^*^ to rhodopsin occurs. On the other hand, if it hits M^*^ after it has inactivated, no photoconversion can occur. They found that M^*^ inactivation is very rapid (τ ∼ 20 ms) under physiological conditions and highly Ca^2+^-dependent, being ∼ 10-fold slower in Ca^2+^-free solutions. Moreover, they found that this process requires calmodulin (CaM) and NINAC, because mutations in *cam* and *ninaC* genes essentially eliminate the Ca^2+^dependence. They proposed that under low-Ca^2+^ conditions, Arr2's within microvilli are bound to NINAC hindering their access to M^*^. Following Ca^2+^ influx, CaCaM binds to NINAC, causing NINAC to release Arr2, which can now diffuse and inactivate M^*^. They argued that this strategy of Ca^2+^ acting via CaM and NINAC to accelerate Arr2-M^*^ binding promotes “quantum efficiency, temporal resolution, and fidelity of visual signaling.”

Considering the multifunctional nature of the NINAC protein, it would not be surprising if NINAC performed many of its functions through interacting protein partners. Recently, such a NINAC-interacting protein has been identified, Retinophilin (Retin/RTP), a poorly characterized protein conserved from flies to humans. Retin/RTP is a retinal phosphoprotein, which is phosphorylated in the dark and dephosphorylated by light, and is identical to the 23-kDa phosphoprotein first identified in two-dimensional (2-D) gel analysis (Matsumoto & Pak, 1984). Two groups have shown recently that this protein is highly expressed in the rhabdomeres and associates with NINACp174, and this interaction is mutually required for the stability of both proteins (Mecklenburg et al., 2010; Venkatachalam et al., 2010). Mecklenburg et al. (2010) showed that mutants in this gene display high rates of spontaneous dark noise, as do *ninaC* mutants (Hofstee et al., 1996), leading to the suggestion that the role of RTP is to suppress random dark noise to promote signaling fidelity (Mecklenburg et al., 2010). Venkatachalam et al. (2010), on the other hand, focused on the age-dependent slow termination of light response in *retin/rtp* mutants, which seems to parallel the loss of eye-enriched protein kinase C (PKC) (INAC) (see Section IIB1). They proposed that loss of Retin/RTP leads in turn to instability and reductions in levels of p174, the scaffold protein INAD (see Section IIB2), and ePKC, and that this ePKC decline underlies the slow termination defect.

### B. The *ina* Mutants

Unlike the *nina* mutants, the rhodopsin levels are normal in most *ina* mutants. In the discussion of mutant phenotypes (Section ID), we noted certain similarities in key features of the ERG between the *ina* mutants and the *trp* mutants of the phototransduction channel, TRP. The *ina* mutant ERGs tend to behave as though they were milder versions of *trp* ERG, although some *ina* mutants, such as *inaF*^P106x^, exhibit phenotypes as strong as that of a null *trp.* The cellular and molecular functions that have been uncovered for the INA proteins to date are diverse. Nevertheless, one common feature that these proteins share is that they are all somehow related to TRP channel functions. Thus, for example, INAC is an eye-enriched PKC whose major substrate is TRP, INAE is a potential key enzyme in the production of messenger(s) to TRP, and INAF is required for the normal expression (and perhaps function) of TRP As for INAD, it orchestrates the formation of a supramolecular signaling complex whose key member is TRP. We now discuss each of the *ina* genes in more detail.

#### 1. *inaC*

The first *inaC* mutants were isolated before the PDA mutant screening protocol was put in place in 1975 because their slow response deactivation phenotype is detectable even without this protocol. However, their identification as *ina* mutants came much later using the protocol.

The *inaC* gene encodes an eye-enriched protein kinase C(ePKC)(Smith et al., 1991), which is expressed primarily in the rhabdomeres (Schaeffer et al., 1989; Smith et al., 1991). It exhibits 53% identity to human PKCβ1 and contains C1 and C2 domains for binding diacylglycerol (DAG) and Ca^2+^, respectively (Shieh et al., 2002).

As to be discussed in the next section (Section IIB2), some of the key components of the *Drosophila* phototransduction cascade are arranged in supramolecular signaling complexes formed by the scaffold protein, INAD, presumably to facilitate speed and efficiency of signaling. Along with PLC_β_ (NORPA), a key activator of the transduction cascade, and TRP, the transduction channel, ePKC is a core member of this signaling complex (Huber et al., 1996; Shieh & Zhu, 1996; Chevesich et al., 1997; Tsunoda et al., 1997). This fact alone suggests its importance in phototransduction, although details of its function still remain to be elucidated.

Null or strong mutants in this gene exhibit a severe defect in response termination (deactivation) (Ranganathan et al., 1991; Smith et al., 1991) and adaptation (Hardie et al., 1993). The deactivation defect is traceable to slow termination of quantum bumps, responses to single photons. In whole-cell recordings, these decay as current noise of unknown origin undergoing damped oscillations (Hardie et al., 1993; Henderson et al., 2000). The TRP channel protein is a major target of INAC-mediated phos-phorylation (Huber et al., 1998; Liu et al., 2000), and proper termination of light response depends at least in part on TRP phosphorylation, since transgenic flies lacking the INAC-dependent phosphorylation site, Ser982 of TRP, display slow response termination (Popescu et al., 2006; see Section IIB2). Other reported substrates of INAC include the scaffold protein INAD (see Section IIB2) (Huber et al., 1996, 1998; Liu et al., 2000) and NINAC myosin III (Li et al., 1998). Phosphorylation of these proteins by INAC has also been implicated in proper termination of light response (Huber et al., 1996; Li et al., 1998).

#### 2. inaD

The first *inaD* mutant, *inaD*^P215^, isolated in August 1974, was one of the first PDA-defective mutants to be isolated. It was the only mutant available in this gene at the time and formed the basis of much of the early work on *inaD*.

The *inaD* gene was cloned and sequenced by Shieh and Niemeyer (1995). The encoded protein was found to be a scaffold protein consisting of five protein-binding PDZ motifs (Postsynaptic Density 95, Discs Large, Zona Occludens 1) (Tsunoda et al., 1997) and to nucleate the formation of a signaling complex by binding several key components of the phototransduction cascade, TRP, NORPA (PLC_β_), and INAC (ePKC) (Huber et al., 1996; Shieh & Zhu, 1996; Chevesich et al., 1997; Tsunoda et al., 1997) in stoichiometric ratios (Huber et al., 1996). INAD is required for the stability and normal localization to the rhabdomeres of the other three proteins (Chevesich et al., 1997; Tsunoda et al., 1997). The complex between INAD and the two soluble proteins, NORPA and INAC, is preformed before the complex reaches the rhabdomeres (Tsunoda et al., 2001). The relationship between INAD and TRP, however, is mutual. Not only does TRP require INAD for its stability and rhabdomeral localization but also INAD needs TRP for the same. Thus, INAD rhabdomeral localization is severely disrupted in *trp* null mutants or in transgenic flies expressing TRP with deleted INAD binding site, resulting in the destruction of the entire signaling complex (Li & Montell, 2000; Tsunoda et al., 2001). The mutual requirement between TRP and INAD is not for targeting of either protein to the rhabdomeres but for longterm retention in the rhabdomeres. That is, either protein can reach the rhabdomeres independently of the other but does not remain there without the other.

Other reported binding partners of INAD include TRPL, rhodopsin (Rh1), NINAC, and calmodulin (CaM) (Xu et al., 1998; Wes et al., 1999). These do not depend on INAD for rhabdomeral localization, and the binding is thought to be dynamic. Nevertheless, the INAD binding site in NINAC was reported to target a heterologous protein to the rhabdomeres (Wes et al., 1999). Since then, the immunophilin, FKBP59 (Goel et al., 2001), and INAF (Cheng & Nash, 2007; see Section IIB4) have also been shown to bind INAD by Western blotting of head extracts. A NINAC-binding protein, retinophilin (Retin/RTP) interacts with INAD indirectly through NINAC (see Section IIA4), and this interaction is reported to be required for the long-term stability of INAD (Venkatachalam et al., 2010). However, Mecklenburg et al. (2010) did not find any noticeable difference in the amount of INAD in their *retin/rtp* mutant.

A number of functions have been ascribed to the INAD scaffolding of signaling proteins. One of the more obvious is the rhabdomeral localization of the key signaling proteins in high concentrations and stoichiometric ratios and the preservation of their stability. The preassembly of the INAD-NORPA-INAC complex would help ensure that the assembled proteins are in the proper composition and correct stoichiometry (Tsunoda et al., 2001). In addition, it has been argued that a major function of INAD complexes is to promote speed and efficiency of transduction by bringing key proteins in close proximity to each other (Huber et al., 1998). INAD and TRP have been reported to be targets of INAC (ePKC)-dependent phosphorylation (Huber et al., 1998; Liu et al., 2000). Thus, the INAD scaffolding would bring the transduction channel, TRP, into the immediate vicinity of its activator, PLC_β_, and its potential regulator, ePKC. Popescu et al. (2006) showed that TRP is phosphorylated by ePKC at Ser^982^ both *in vitro* and *in vivo* and that this phosphorylation depends on INAD *in vitro*. Moreover, mutation of this site leads to slow response termination kinetics, in support of INAD's role in promoting speed. Other evidence in support of the above argument includes the following: (1) genetic elimination of INAC (ePKC) results in slow response termination that is observed at the bump level (Hardie et al., 1993; Henderson et al., 2000) (see Section IIB1 on *inaC*) and (2) a similar phenotype is also observed in *inaD*^P215^ mutants (Henderson et al., 2000), in which INAD-TRP binding is defective (Shieh & Niemeyer, 1995; Shieh & Zhu, 1996). However, it has also been reported that transgenic flies expressing TRP with deleted INAD-binding site show normal response kinetics (Li & Montell, 2000).

Recent evidence suggests that the INAD scaffold may have a dynamic role in signaling in addition to its role as a scaffold for the formation of signaling complexes. Mishra et al. (2007) carried out crystal structure studies of the recombinant peptide of INAD PDZ-5, which is a PLC-binding domain (Shieh et al., 1997; van Huizen et al., 1998). Their studies suggested that PDZ-5 exists in two redox-dependent conformations—a reduced state maintained in the dark and an oxidized state switched on by light-dependent formation of an intramolecular Cys-Cys disulfide bond (Mishra et al., 2007). The formation of the disulfide bond would distort the PLC-binding groove to potentially regulate signaling. Mutants in which the formation of the disulfide bond is disrupted exhibit defects in response termination, the bump refractory period, and escape behavior. Insights into the potential mechanism of light-dependent cycling of the redox state of PDZ5 were provided by Liu et al. (2011). They showed that whereas isolated PDZ5 is stable in the oxidized state, its interaction with the neighboring PDZ4 to form a PDZ4-5 supramolecule allosterically raises its redox potential to lock it in the reduced state. A light stimulus uncouples the PDZ4-5 interactions to switch PDZ5 to the oxidized state. The uncoupling is suggested to be mediated by acidification of the microvillar microenvironment by protons released during light-mediated hydrolysis of PIP_2_ by PLC_β_ (Huang et al., 2010).

When we started screening for mutants to study phototransduction (review: Pak, 2010), one of the major criticisms of the approach at the time was that phototransduction was much too fast to involve a large number of proteins, as would be the assumption in large-scale forward genetic mutant screens. If signal transfer from an activated molecule to its downstream target occurs simply by diffusion and random collisions, it would indeed take a long time to get through multistep processes. However, little was known about the microenvironment in which phototransduction takes place. As we have related above, the study of mutants generated in the screen have now provided a reasonable and likely answer to what was then a vexing conundrum.

#### 3. inaE

The *inaE* gene encodes a *sn*-1–specific diacylglycerol lipase (DAGL) expressed abundantly in photoreceptors in addition to other tissues (Leung et al., 2008). It was the first *Drosophila* DAGL to be characterized. The importance of *inaE*-encoded DAGL (INAE) lies in its potential involvement in the activation of TRP/TRPL channels. The mechanism(s) of activation of *Drosophila* phototransduction channels is not known and is currently under active investigation (see reviews: Katz & Minke, 2009; Hardie, 2011). At least five models of TRP/TRPL channel activation have been proposed. We will discuss two of these here that are directly relevant to INAE.

**Figure 4:**
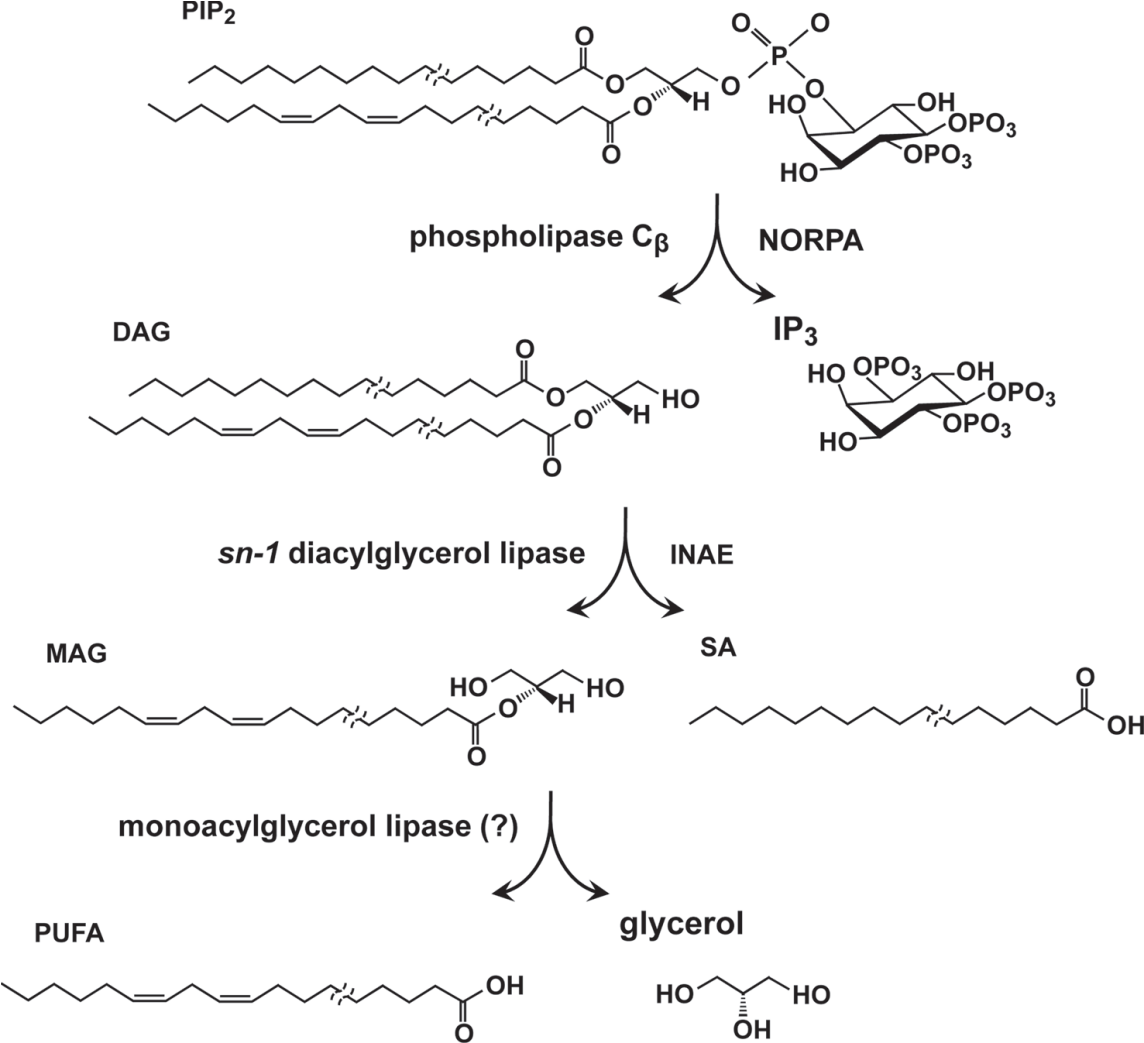
PIP_2_ hydrolysis in the phototransduction cascade. Upon light stimulation, PIP_2_ is hydrolyzed by PLC_β_ (NORPA) to yield diacylglyceorol (DAG) and inositol trisphosphate (IP_3_). DAG is hydrolyzed by the *sn*-1 DAG lipase, INAE, to yield 2-monoacylglycerol (2-MAG) and free fatty acid liberated from the *sn*-1 position of the DAG substrate. 2-MAG is hypothesized to be further hydrolyzed by MAG lipase to yield polyunsaturated fatty acid (PUFA) and glycerol. Note that *Drosophila* photoreceptor PIP_2_ is likely to be composed of acyl chains of varying lengths and that the *sn*-1 and -2 positions are preferentially occupied by the saturated and unsaturated acyl chains, respectively.

The *Drosophila* phototransduction cascade generates two potential second messengers to the channels, inositol 1,4,5-trisphosphate (IP_3_) and diacylglycerol (DAG), from the hydrolysis of phosphatidylinositol 4,5-bisphosphate (PIP_2_) by PLC_β_ (NORPA) (Figure 4). IP_3_ has been excluded as a candidate messenger by genetic evidence (Acharya et al., 1997; Raghu et al., 2000a). DAG and its hydrolysis products, polyunsaturated fatty acids (PUFAs), have both been proposed as agents of channel activation. Exogenous application of PUFAs has been shown to activate TRP/TRPL channels in intact photoreceptors, heterologous expression systems (Chyb et al., 1999), and more recently inside-out patches of rhabdomeral microvillar membrane (Delgado & Bacigalupo, 2009). However, no genetic or biochemical evidence exists to support the observations. DAG's proposed role as an excitatory agent is largely based on the observation that in *rdgA* mutants TRP/TRPL channels are constitutively active (Raghu et al., 2000b). The *rdgA* mutation blocks the conversion of DAG to phosphatidic acid (PA) (the first step in resynthesis of PIP_2_) and thus is expected to raise the basal level of DAG. If DAG is excitatory to the channel, a rise in its basal levels would activate TRP/TRPL channels and thus could explain the constitutive activity of TRP/TRPL channels. However, since DAG would be hydrolyzed by DAG lipase, *rdgA* mutations are expected to raise the basal levels not only of DAG but also of its hydrolysis products (Figure 4). Thus, *inaE*, encoding DAGL, could be important in providing insights into the potential roles of DAG vs. its metabolites (Figure 4).

Strong mutations generated in *inaE* were lethal and had to be examined as eye mosaics, in which only the eye tissues were homozygous for the mutation (Leung et al., 2008). These mutations severely reduced the responses of photoreceptor to light in an allele-dependent manner, leading to the suggestions that hydrolysis products of DAG generated by DAGL activity, not DAG itself, are responsible for TRP channel excitation. This work represented a first step in an attempt to understand lipid regulation of TRP channels and raised a number of questions. The first of these is that the INAE protein localizes primarily to submicrovillar cisternae and photoreceptor cell bodies with just traces of staining in the rhabdomeres (Leung et al., 2008). The second problem is that INAE is a type *sn*-1 DAGL. It releases 2-monoacylglycerol (2-MAG) and mostly saturated fatty acid from DAG and does not release PUFA directly (Figure 4). PUFA can be released from 2-MAG by the action of MAG lipase (MAGL) in a subsequent reaction. However, invertebrate MAGLs are a poorly understood class of lipases, and no direct orthologs of mammalian or bacterial MAGL appear to be present. Using a combined bioinformatic, biochemical, and molecular approach, we are now focusing on a candidate MAGL gene with a desired expression profile.

A new model of phototransduction has been put forward by Huang et al. (2010). They have proposed that the two consequences of light-induced hydrolysis of PIP_2_ by PLCβ, namely, the reduction in the levels of PIP_2_ (and other phosphoinositides) and the release of protons, may act combinatorially to activate the TRP/TRPL channels. In support of this hypothesis, they showed, using pH indicator dyes, a light-dependent acidification of ∼0.1 pH unit in photoreceptors in the time scale comparable to that of phototransduction. Moreover, following thorough depletion of PIP_2_, TRP/TRPL channels could be activated by the metabolic poison, 2,4-dinitrophenol, which is a protonophore. Their hypothesis is novel and striking and merits serious consideration. Its long-term significance is not yet clear.

#### 4. *inaF*

Unlike the other mutants discussed herein, *inaF* mutants were not the products of the original EMS (ethyl methanesulfonate) mutagenesis of the Pak laboratory (review: Pak, 2010). Instead, they were isolated in P element mutagenesis of an unrelated gene long after the early mutagenesis efforts had ceased (Li et al., 1999). The *inaF* gene encodes a small, eye-enriched protein, initially thought to be encoded by a 241-aa ORF (open reading frame) in the second exon of the transcript. Subsequently, Cheng and Nash (2007) showed that the protein critical to *inaF* function (designated INAF-B) is encoded by an 81-aa ORF in the first exon of the transcript. The null *inaF* phenotype includes greatly reduced levels of the TRP channel protein (∼5–10%), without any effect on other phototransduction proteins tested, and ERG responses closely resembling those of *trp* null mutants (Li et al., 1999). The mRNA levels, however, are unaffected by *inaF* mutations (Cheng & Nash, 2007). The INAF-B protein is a single-pass, integral membrane protein that colocalizes and interacts with TRP in the rhabdomere (ibid.). The interaction between INAF-B and TRP is required for their mutual survival and persists even in a *inaD* null mutant background. INAF-B also interacts with the scaffold protein INAD, suggesting that it may be a member of the signaling complex formed by INAD (see Section IIB3 above). The INAF-B coding sequence contains a 32-aa motif (*inaF* motif) conserved from flies and worms to fish and humans (Cheng & Nash, 2007). The TRPL channels appear to function normally in *inaF*, and the residual TRP is still localized to the rhabdomeres (Li et al., 1999). Li et al. (1999) assessed the effects of *inaF* null on the ERG waveform, the refractory period, and the ability to rescue the degeneration phenotype of the constitutively active *trp* mutant (*Trp*^P365^/+) and compared them to the effects of comparable depletions in the amount of TRP protein. They argued that the *inaF* phenotype could not be explained by the reduction in the amount of the TRP protein alone and suggested that the INAF protein may have a role in TRP channel function. The subsequent biochemical findings on INAF-B by Cheng and Nash tend to reinforce this suggestion. However, INAF cellular function remains unknown.

## ANECDOTAL STORY AND CONCLUDING REMARKS

At times a seemingly ordinary event can profoundly alter the course of development of a field. This is one such story. In the spring of 1984, one of us was on sabbatical at the Department of Biological Chemistry, Harvard Medical School. Gerald Rubin had once been on the faculty at Harvard Medical School. In the spring of 1984, he came back to Harvard Medical School as a guest speaker. Rubin sought out Pak to express his interest in the phototransduction mutants Pak had been isolating. Rubin explained that he had just moved to University of California at Berkeley and would be organizing a seminar course on *Drosophila* neurogenetics the following semester and would like to invite Pak as one of the speakers. Pak was surprised that Rubin knew about his mutants at all. As best as Pak can recall, Rubin called him in the fall of 1984 at Biocentrum, University of Basel, where he was doing the second half of his sabbatical, to arrange for his visit to Berkeley the following March. In addition, Rubin asked for the following *nina* and *ina* mutants: *ninaA*, *B*, *C*, *D*, and *E* and *inaA*, *B*, *C*, and *D*. Fairly detailed characterizations of *ninaA*, *B*, *C*, *D*, and *E* mutants were published in a symposium volume (Stephenson et al., 1983). As for the *ina* mutants, except for brief mentions of the fact that some of them were being isolated in a review and a symposium volume (Pak, 1979; Pak et al., 1980), nothing had yet been published on any of them; nevertheless, Pak sent all the requested mutants. Pak went to Berkeley in March 1985 and gave two talks introducing the audience to the field of *Drosophila* phototransduction and the mutants he had been isolating. Subsequently, two of Rubin's talented postdoctoral associates, Craig Montell and Charles Zuker, decided to go into the field of *Drosophila* phototransduction. Glancing through the references makes it clear, these two investigators and their associates and students went on to make major contributions to this field.

Perusal through the References also makes it clear that many other talented investigators from all over the world also contributed to the progress in this field. Although the *nina*/*ina* mutants may be the common thread running through this story, the story is really about the contributions of all these investigators.

In this review, we have attempted to provide a reasonably comprehensive view of the PDA-defective mutants starting with the rationale for the PDA-based mutant screening strategy to summaries of knowledge gained from the mutants isolated through this strategy. The PDA-based strategy turned out to be a surprisingly effective and efficient means of identifying genes important in photoreceptor function. Almost every gene that has been identified turned out to be interesting, and the importance of the proteins encoded by many of these genes in cellular signaling is already being recognized, with ramifications of their significance extending far beyond the field of photoreceptor function. Many of the proteins encoded by the *nina*/*ina* genes so far studied turned out to be the first member of their class to be identified. Thus, NINAE was the first invertebrate opsin to be identified and characterized; NINAA was the first chaperone protein for a GPCR (G protein–coupled receptor) to be identified; NINAC was the first class III myosin to be identified; NINAB was the first invertebrate β-carotene oxygenase to be identified; and INAD led to the discovery of the first supramolecular signaling complex in sensory transduction cascades. The discovery of the INAD-based signaling complexes promises to be particularly important in our understanding of sensory signaling cascades in general.

Although much has been achieved, we have not yet fully tapped the potential of the *nina*/*ina* mutants. As may be seen in Table 1, a sizable number of *nina*/*ina* mutants remain uncharacterized. Moreover, the PDA-based mutagenesis was never carried out to saturation. There are many more *nina* and *ina* mutants to be identified and isolated. Our expectation is that these mutants will continue to contribute to our understanding of cellular signaling for some time to come.

## Footnotes

1 We will use the term “inactivation” to refer to the decay of response or desensitization of cell during stimulus. The term “deactivation” will be used to refer to response decay at the end of stimulus.

2 In the days before the development of efficient P element–mediated mutagenesis, P element mutagenesis was performed by crossing P strain males with M strain females to induce hybrid dysgenesis, a high rate of mutation in germ line cells (e.g., review: Engels, 1996).

3 Since all *nina* mutants were isolated on the basis of alterations in the PDA generated in R1–6 cells, these mutations only detect defects in R1–6 cells. R7/8 cells might or might not also be affected.
